# Beyond the synapse: Bassoon mutations drive epilepsy through Astrocyte–Neuron crosstalk

**DOI:** 10.1016/j.neurot.2025.e00753

**Published:** 2025-09-30

**Authors:** Xindi Li, Ronald P. Hart, Zhiping P. Pang

**Affiliations:** a Center for NeuroMetabolism, Child Health Institute of New Jersey, Rutgers University, Robert Wood Johnson Medical School, United States; b Department of Neuroscience and Cell Biology, Rutgers University, Robert Wood Johnson Medical School, United States; c Department of Cell Biology and Neuroscience, Rutgers University, United States

**Keywords:** Active zone, Synapse, Neuron-glia interactions, Seizure, Reactive astrocyte

The *BSN* gene, which encodes the presynaptic scaffolding protein Bassoon, has been increasingly recognized for its association with epilepsy [1] and broader neurodevelopmental disorders [2]. Recent cohort studies have shown that individuals carrying pathogenic *BSN* variants frequently experience seizures during infancy or early childhood, which coincides with the peak expression of Bassoon in the developing brain [3,4]. The clinical presentations are diverse, including febrile seizures, generalized tonic-clonic seizures, and focal seizures [3,4]. Notably, the severity and specific clinical features appear to vary depending on the type of *BSN* variant, with missense mutations, truncating variants, and biallelic alterations each linked to distinct phenotypes and prognoses [3]. These findings highlight the genetic heterogeneity of *BSN*-related epilepsy and underscore the importance of understanding genotype-phenotype correlations to guide diagnosis and prognosis.

Bassoon is a large presynaptic scaffold protein (∼420 ​kDa) localized at the active zone of neurons, where it is specialized for synaptic vesicle docking and fusion [5]. Its N-terminal domain associates with the trans-Golgi network and interacts with Piccolo to facilitate the assembly and trafficking of active zone precursor vesicles [6]. At the synapse, the C-terminal region serves as a molecular hub, binding to RIMs, Munc13s, ELKS/CAST proteins, and voltage-gated Ca^2+^ channels, thereby coordinating vesicle docking, priming, and Ca^2^-triggered neurotransmitter release [7]. Loss of functional Bassoon in mutant mice results in hyperexcitability and spontaneous seizures, highlighting its essential role in synaptic homeostasis and its relevance to epilepsy [8]. Interestingly, recent single-cell transcriptomic datasets (e.g., GSE255968, GSE280481) have detected *BSN* expression not only in neurons but also in astrocytes. This observation raises the intriguing possibility that Bassoon may play a role in astroglial regulation of the synaptic microenvironment, although its function in astrocytes remains largely uncharacterized.

Astrocytes, the most abundant glial cells in the central nervous system, are essential for maintaining neuronal homeostasis and regulating neuronal excitability by providing metabolic support, buffering ions, and recycling neurotransmitters. Evidence suggests that astrocytic dysfunction could promote seizure susceptibility and the development of epilepsy [9,10]. In temporal lobe epilepsy, reactive astrocytes accumulate excess lipids, giving rise to a distinct subtype termed lipid-accumulated reactive astrocytes (LARAs), which are associated with increased neuronal excitability and seizure severity [11]. This lipid accumulation is also exacerbated by impaired mitochondrial fatty acid β-oxidation, indicating that intrinsic astrocytic metabolic deficits can actively drive epileptogenesis [12]. Beyond lipid metabolism, astrocytes play an important role in regulating neuronal excitability and maintaining network stability by buffering extracellular potassium and clearing excess glutamate. Dysfunction of the astrocytic Kir4.1 channel disrupts K^+^ and glutamate homeostasis, contributing to neuronal hyperactivity in both human epilepsy and animal models [13,14]. Collectively, these findings suggest astrocytic lipid dysregulation and impaired ion and neurotransmitter homeostasis could contribute to seizure generation, highlighting astrocytes as promising therapeutic targets alongside neurons.

In this issue of Neurotherapeutics, *Chen* *et al.* uncover previously unrecognized roles for Bassoon in astrocytic metabolism, reframing Bassoon as not only a synaptic organizer in neurons but also a metabolic regulator in glia. Using CRISPR-edited iPSC lines carrying pathogenic *BSN* variants, the authors differentiated astrocytes and validated the expression of key astrocytic markers (GFAP, ALDH1L1, S100B), as well as confirming the loss of Bassoon protein. Upon kainic acid (KA) stimulation, *BSN*-mutant astrocytes exhibited marked accumulation of free cholesterol, particularly in 5672insCG frameshift iPSC line-derived astrocytes. Arginase activity, a key regulator of arginine and lipid metabolism, was significantly reduced in homozygous M1903V and 5672insCG astrocytes compared to their isogenic controls. Arginase deficiency has previously been shown to promote tissue lipid accumulation via enhanced de novo lipogenesis independent of systemic metabolic changes [15], suggesting that *BSN* mutations may drive a cholesterol–arginase imbalance as a mechanistic contributor to epilepsy.

*Chen* *et al.* next investigated how dysfunctional astrocytes might influence neuronal activity. They found that *BSN*-mutant astrocytes exhibited reduced expression of Kir4.1 and TREK-1 channels. This impairs K^+^ buffering, leading to extracellular potassium accumulation and subsequent neuronal depolarization, increased neuronal excitability, and heightened seizure susceptibility. Mechanistically, Kir4.1 dysfunction may arise from both lipid-induced membrane rigidity and inflammatory signaling, linking metabolic stress to ionic imbalance and hyperexcitability. Additionally, *BSN*-mutant astrocytes exhibited hyperphosphorylation of ERK and CREB, indicating an activated inflammatory state. When neurons were co-cultured with these astrocytes, they were exposed to elevated levels of IL-1β, TNF-α, and reactive oxygen species. This inflammatory milieu shifted neuronal survival pathways, increasing pro-apoptotic markers (Bax, cleaved caspase-3) while reducing the survival protein Bcl-2. Functionally, neurons in this environment exhibited heightened excitability, characterized by increased firing frequency, spike amplitude, and intracellular Ca^2+^ levels. Together, these findings demonstrate that lipid accumulation, ion channel dysregulation, and inflammatory signaling in astrocytes can disrupt neuronal excitability, function, and survival, positioning astrocyte-neuron dysfunction as a central driver of *BSN*-related epilepsy.

While this work provides important insights, several limitations should be acknowledged. First, the precise subcellular localization of Bassoon in astrocytes and its mechanistic link to arginase activity and lipid metabolism remain undefined, and it is unclear whether the observed lipid accumulation represents a general astrocytic phenotype or is restricted to reactive astrocytes induced by kainic acid stimulation. Second, all experiments were conducted in a single human iPSC line; whether mutations in *BSN* in other genetic backgrounds or patient-specific iPSCs share the same pathology remains to be elucidated. For example, additional genomic variants elsewhere in the genome are likely to modulate astrocytic dysregulation produced by Bassoon variants. Third, the study focused primarily on astrocytic mechanisms; the direct cell-autonomous effects of *BSN* mutations in neurons were not examined. It remains unresolved whether neuronal hyperexcitability is driven intrinsically by mutant neurons or arises secondarily from impaired astrocyte–neuron interactions.

In summary, *Chen* *et al.* provide compelling evidence that *BSN* mutations in human astrocytes drive lipid accumulation, ion channel dysfunction, and neuronal hyperexcitability. By linking metabolic stress to ionic imbalance and excitability, the study also identifies the cholesterol-arginase axis and glial lipid handling pathways as potential therapeutic targets for *BSN*-related epilepsy.

## Author’s contribution

X.L. wrote the commentary under the supervision of R.P.H. and Z. P.

## Declaration of competing interest

There is no conflict of interest to declare.

## References

[1] Skotte L., Fadista J., Bybjerg-Grauholm J., Appadurai V., Hildebrand M.S., Hansen T.F. (2022). Genome-wide association study of febrile seizures implicates fever response and neuronal excitability genes. Brain.

[2] Kaladiyil A.P., Kukkle P.L. (2025). Bassoon, presynaptic scaffolding protein: narrative review in health and disease. Eur J Neurosci.

[3] Ye T., Zhang J., Wang J., Lan S., Zeng T., Wang H. (2023). Variants in BSN gene associated with epilepsy with favourable outcome. J Med Genet.

[4] Guzman S.G., Ruggiero S.M., Ganesan S., Ellis C.A., Harrison A.G., Sullivan K.R. (2025). Variants in BSN, encoding the presynaptic protein Bassoon, result in a distinct neurodevelopmental disorder with a broad phenotypic range. Am J Hum Genet.

[5] Garner C.C., Kindler S., Gundelfinger E.D. (2000). Molecular determinants of presynaptic active zones. Curr Opin Neurobiol.

[6] Gundelfinger E.D., Reissner C., Garner C.C. (2015). Role of Bassoon and Piccolo in assembly and molecular organization of the active zone. Front Synaptic Neurosci.

[7] Chua J.J. (2014). Macromolecular complexes at active zones: integrated nano-machineries for neurotransmitter release. Cell Mol Life Sci.

[8] Ghiglieri V., Picconi B., Sgobio C., Bagetta V., Barone I., Paillè V. (2009). Epilepsy-induced abnormal striatal plasticity in Bassoon mutant mice. Eur J Neurosci.

[9] Chen Y., Hu J., Zhang Y., Peng L., Li X., Li C. (2026). Epilepsy therapy beyond neurons: unveiling astrocytes as cellular targets. Neural Regen Res.

[10] Purnell B.S., Alves M., Boison D. (2023). Astrocyte-neuron circuits in epilepsy. Neurobiol Dis.

[11] Chen Z.P., Wang S., Zhao X., Fang W., Wang Z., Ye H. (2023). Lipid-accumulated reactive astrocytes promote disease progression in epilepsy. Nat Neurosci.

[12] Su Y., Tang M., Wang M. (2024). Mitochondrial dysfunction of astrocytes mediates lipid accumulation in temporal lobe epilepsy. Aging Dis.

[13] Kinboshi M., Ikeda A., Ohno Y. (2020). Role of astrocytic inwardly rectifying potassium (Kir) 4.1 channels in epileptogenesis. Front Neurol.

[14] Sicca F., Ambrosini E., Marchese M., Sforna L., Servettini I., Valvo G. (2016). Gain-of-function defects of astrocytic Kir4.1 channels in children with autism spectrum disorders and epilepsy. Sci Rep.

[15] Navarro L.A., Wree A., Povero D., Berk M.P., Eguchi A., Ghosh S. (2015). Arginase 2 deficiency results in spontaneous steatohepatitis: a novel link between innate immune activation and hepatic de novo lipogenesis. J Hepatol.

